# Relationships between blood pressure indicators and fluid biomarkers of brain aging in functionally intact older adults

**DOI:** 10.1186/s13195-025-01731-9

**Published:** 2025-04-21

**Authors:** Anna M. VandeBunte, Bailey L. Ortiz, Emily W. Paolillo, Rowan Saloner, Valentina Diaz, Shubir Dutt, Claire J. Cadwallader, Coty Chen, Argentina Lario Lago, Julio C. Rojas, Brandon Chan, Isabel Sible, Joel H. Kramer, Kaitlin B. Casaletto

**Affiliations:** 1 Memory and Aging Center, Department of Neurology, University of California, San Francisco, 675 Nelson Rising Lane, Suite 190, San Francisco, CA 94158 USA; 2 Palo Alto University, Palo Alto, CA 94304 USA

**Keywords:** Cardiovascular health, Astrocytic activation, Axonal breakdown, Blood pressure, Pulse pressure

## Abstract

**Background:**

Dementia risk is significantly shaped by cardiovascular health, with elevated blood pressure emerging as a key risk factor for adverse brain aging. Blood biomarkers such as pTau181, Aβ42/40, NfL, and GFAP have improved our understanding of dementia pathophysiology, however, few studies have explored how specific blood pressure metrics relate to biomarker levels, which could inform personalized dementia prevention strategies as these biomarkers move into clinic. We examined how different blood pressure metrics associated with molecular markers of astrocytic activation (GFAP), neuronal axon breakdown (NfL), and Alzheimer’s disease pathobiology (pTau181, Aβ42/40) in plasma.

**Methods:**

109 functionally intact (Clinical Dementia Rating Scale = 0) older adults completed blood draws with plasma assayed for Aβ42/40, GFAP, NfL, and pTau181 (Quanterix Simoa) and in-lab blood pressure quantification. Blood pressure metrics included diastolic blood pressure, systolic blood pressure, and pulse pressure (systolic minus diastolic). Separate regression models evaluated plasma biomarkers as a function of each blood pressure metric, adjusting for age and biological sex. Interaction models tested whether relationships between blood pressure metrics and plasma biomarkers differed by sex, age, or *APOE*-ε4 status.

**Results:**

With the exception of Aβ42/40, higher pulse pressure related to higher levels of all plasma biomarkers examined (pTau181, NfL, GFAP). Additionally, higher systolic blood pressure related to higher pTau181, while diastolic blood pressure did not meaningfully associate with any biomarker. Interaction models revealed a significantly stronger relationship between elevated pulse pressure and higher GFAP concentrations in females compared to males, as well as a significantly stronger association between elevated pulse pressure and lower Aβ42/40 plasma concentrations in *APOE*-ε4 carriers compared to non-carriers.

**Conclusions:**

Our findings suggest that elevated pulse pressure, and to a lesser extent systolic blood pressure, are associated with increased Alzheimer’s disease and neurodegenerative (axonal and astrocytic health) biology among typically aging adults. These associations underscore the importance of blood pressure management, particularly pulse pressure, for reducing dementia risk. Cardiovascular health may be incorporated with biomarkers to further personalize dementia prevention and management strategies.

**Supplementary Information:**

The online version contains supplementary material available at 10.1186/s13195-025-01731-9.

## Background

Dementia is among the top 10 leading causes of death worldwide, with an estimated 55.2 million individuals affected [1]. Yet, up to 45% of dementia cases are attributable to modifiable risk [2]. The Lancet Commissions *Dementia Prevention*,* Intervention*,* and Care* report highlighted 14 key modifiable factors to target dementia risk and promote healthy brain aging [2]. Notably, half of these factors can be directly tied to cardiovascular and cardiometabolic health (i.e., high LDL cholesterol, physical inactivity, diabetes, smoking, hypertension, obesity, excessive alcohol use). Previous literature has linked poor systemic cardiovascular health during midlife (e.g., hypertension, heart disease, stroke) with increased risk of dementia later in life [3, 4]. Among cardiovascular risk factors, blood pressure consistently emerges as a key risk indicator driving both cardiovascular and brain health outcomes [5–8]. For instance, midlife hypertension management is estimated to decrease the later life dementia risk by 2% [2]. A meta-analysis also found that elevated blood pressure related to lower brain volumes in regions impacted by Alzheimer’s disease (AD) (e.g., hippocampus), suggesting a potential direct role in neurodegeneration [9]. Moreover, recent data from the SPRINT-MIND trial showed that intensive blood pressure control reduces risk of mild cognitive impairment [6]. Thus, it is increasingly clear that blood pressure is an important modifiable target for dementia prevention.

Blood pressure can be assessed using different metrics, including systolic pressure (the peak pressure during heartbeats), diastolic pressure (the minimum pressure between beats), and pulse pressure ([systolic blood pressure - diastolic blood pressure], which reflects arterial stiffness) [10]. Specifically, elevated systolic blood pressure and pulse pressure have been linked to accelerated brain aging and increased risk of cerebral small vessel disease [11–13]. Conversely, lower diastolic blood pressure in the absence of elevated systolic blood pressure has also been associated with adverse cognitive outcomes [14]. Although inadequate blood pressure control is associated with negative effects on cognitive health and an increased risk of dementia, our understanding of which blood pressure metrics may be most sensitive to brain health is limited. Greater understanding of which blood pressure metrics are most important for brain aging could inform more precise recommendations.

Detection of dementia risk and understanding of dementia pathophysiology in humans has significantly advanced with the utilization of blood biomarkers. Biomarkers such as phosphorylated tau isoforms (pTau), beta amyloid isoforms (Aβ), neurofilament light chain (NfL), and glial fibrillary acidic protein (GFAP) are among the most robust, widely studied, and sensitive indicators of dementia risk; they are increasingly being used in memory clinics and provide cost-effective, scalable alternatives to gold-standard PET imaging and CSF markers. Plasma pTau181 concentrations are specifically elevated in AD, tracking closely with amyloid-PET and longitudinal cognitive decline, and are thought to reflect levels of amyloid-related tau hyperphosphorylation [15, 16]. Amyloid beta ratios (Aβ42/Aβ40) similarly aim to evaluate amyloid burden due to AD and are associated with greater cognitive decline, though to a lesser degree than pTau analytes [17]. On the other hand, NfL is a nonspecific marker reflecting general neuronal axon degeneration and often considered a marker of biological disease severity in the context of dementia [18, 19]. Finally, GFAP is a marker of astroglial activation and may be sensitive to early amyloid related changes in AD, as well as cerebrovascular injury [20, 21]. Emerging evidence suggests that these plasma biomarkers (GFAP, NfL, pTau181, Aβ42/40) reflect processes of brain aging, even in cognitively healthy individuals, by capturing subtle neurobiological changes linked to neuroinflammation, axonal integrity, cerebrovascular function, and early Alzheimer-disease related pathobiology. For example, higher plasma levels of pTau181 and GFAP in cognitively unimpaired older adults have been associated with future brain atrophy and cognitive decline over a median follow-up of five to six years [22]. Additionally, plasma NfL, GFAP, and pTau181 consistently and strongly increase with age further highlighting their potential as markers of aging-related brain changes [23].

To date, several studies have investigated the relationship between plasma markers of neural health and systemic cardiovascular risk, often using a composite score that includes multiple cardiovascular factors. For instance, the Framingham Risk Score, a common composite metric of cardiovascular burden, has been previously associated with higher blood levels of NfL and markers of AD pathobiology (e.g., beta amyloid 42/40, total tau) [24]. Other studies have demonstrated relationships between cardiovascular composite scores and cognitive decline, independent of fluid biomarkers (e.g., beta amyloid, pTau, and total tau) [25]. Among individual indicators of cardiovascular health, blood pressure has emerged as a key risk factor for dementia. Blood pressure metrics are quick, easy to obtain, and routinely measured during primary care visits, making them an accessible and practical tool for early detection of health risks. However, few studies have explored associations between specific blood pressure measures and fluid biomarkers, limiting our understanding of which blood pressure metrics are most relevant to cognitive and brain aging.

Our aim was to determine which blood pressure metrics (e.g., systolic blood pressure, diastolic blood pressure, pulse pressure) most robustly relate to fluid biomarkers of brain aging in a cohort of functionally intact older adults, including astrocytic activation (GFAP), neuronal axon breakdown (NfL), and AD pathobiology (pTau181; Aβ42/40). We secondarily aimed to examine how person-specific dementia risk factors, such as age, sex, and AD risk gene apolipoprotein ε4 (*APOE*-ε4) carrier status (yes/no), influence the relationships between blood pressure and key biomarkers of brain aging. We hypothesized that males, who are generally more predisposed to cardiovascular disease, will demonstrate stronger associations between elevated blood pressure and fluid biomarker outcomes [26]. Given that neurodegenerative protein deposition typically increases, and vascular health typically decreases with advancing age, we also expected older individuals to show disproportionately stronger adverse relationships between blood pressure and fluid biomarker concentrations [27]. Finally, given the *APOE*-ε4 gene is linked to increased AD risk and cardiometabolic dysfunction, we hypothesized that ε4 carriers would demonstrate stronger associations between blood pressure and all biomarkers evaluated (GFAP, NfL, pTau181, Aβ42/40) [28]. Ultimately, a greater understanding of how distinct metrics of blood pressure relate to biomarkers reflecting early AD related pathobiology and adverse brain aging could inform more precise primary prevention approaches for reducing dementia risk.

## Methods

### Participants

109 functionally intact older adults enrolled in the University of California, San Francisco Memory and Aging Center’s Brain Aging Network for Cognitive Health Study who completed blood pressure quantification and blood draws with plasma assayed for Aβ42/40, GFAP, NfL, and pTau181 via Quanterix Simoa were included in the study (Table 1). All participants underwent comprehensive neurologic and neuropsychological evaluations and were classified as cognitively normal per consensus review and/or a Clinical Dementia Rating Scale of 0, per study partner interview. Participants were excluded from the study if they had a diagnosis of any other major neurological condition (e.g., epilepsy, stroke) or a neurodegenerative disease (e.g., frontotemporal dementia).

**Table 1:** Demographic and clinical characteristics of study sample

	*n*	%(*n*) or M(SD)
Sex, % Female	109	55.04% (60)
Race	109	
White		79.82% (87)
Black		< 1% (1)
Asian		15.60% (17)
Other		4.59% (5)
Age (years)	109	73.2(8.1)
Education (years)	109	17.5(1.9)
Body mass index (BMI)	109	25.4(4.9)
*APOE* Status	96	
* E2/E3*		10.42% (10)
* E2/E4*		1% (1)
* E3/E3*		65.6% (63)
* E3/E4*		21.9% (21)
* E4/E4*		1% (1)
Hypertension, % recent/active history	101	31.68% (32)
Anti-hypertensive medication, % current use	108	34.26% (37)
Cardiovascular indicators		
Systolic blood pressure (BP, mmHg)	109	132.7(16.4)
Diastolic BP (mmHg)	109	74.0(9.6)
Pulse pressure (systolic BP-diastolic BP, mmHg)	109	58.7(14.1)
Plasma biomarkers (pg/mL)		
Amyloid beta 42/40 ratio (Aβ42/40)	107	0.6(0.01)
Glial fibrillary acidic protein (GFAP)	109	167.9(87.5)
Neurofilament light chain (NfL)	109	29.6(14.6)
Tau phosphorylated at threonine-181 (pTau181)	98	3.8(2.1)

Note. *n* = sample size available for each characteristic. *M* = mean, *SD* = standard deviation. *Carriers include individuals who have at least one copy of the ε4 allele

The study was approved by the UCSF Institutional Review Board and is conducted in accordance with the latest Declaration of Helsinki, including written informed consent from all participants.

### Blood pressure indicators

Blood pressure metrics of interest included systolic blood pressure, diastolic blood pressure, and pulse pressure, given previously reported relationships between each of these factors and brain health [29, 30].

A mobile Masimo Root^®^ vital signs monitor (Masimo Co., Irvine, CA, USA) was used to measure participant’s blood pressure during the study visit. Blood pressure (BP) readings were collected by a clinician or study staff following standard procedures. Pulse pressure (systolic BP-diastolic BP) was calculated by subtracting diastolic blood pressure from systolic blood pressure, following previous publications [31]. Normal adult systolic blood pressure readings range from 120 to 130 mmHg, while the target adult diastolic blood pressure reading is less than 80 mmHg (range 60–80 mmHg) [32]. Optimal pulse pressure ranges from approximately 40–50 mmHg [33–35]; values greater than 60 have been associated with increased risk of cardiovascular disease [36].

### Plasma biomarker quantification

Plasma markers of interest included amyloid beta 42/40 ratio (Aβ42/40), phosphorylated tau (pTau181), neurofilament light chain (NfL) and glial fibrillary acidic protein (GFAP).

Venous blood was collected in EDTA-containing tubes, and plasma samples were stored in 0.5 mL aliquots at − 80 C. Blood samples (1 thawing only) were gradually brought to room temperature for analysis. The ultrasensitive HD-X analyzer by Quanterix (Lexington, MA) was used for quantification of proteins. GFAP, Aβ42, Aβ40, and NfL were measured via commercially available multiplex single molecule arrays (Simoa, Quanterix Neurology 4-Plex A), while pTau181 was measured using a single analyte assay (Simoa, Quanterix). All analyses were performed in duplicate, according to manufacturer’s published protocols, by investigators blinded to sample identity. Samples with coefficients of variance > 20% were excluded from analyses, this included 11 for pTau181 and none for the other markers. Final data were also examined for outliers, and samples less than Q1–3*IQR or greater than Q3 + 3*IQR were also excluded (*n* = 2 for Aβ42/40).

### APOE genotyping

Standard procedures were employed to extract genomic DNA from peripheral blood (Gentra PureGene Blood Kit, Qiagen). TaqMan or Sequenom were used to perform the genotyping. *APOE* genotyping (rs429358 and rs7412) was achieved using the TaqMan Allelic Discrimination Assay, which was conducted on an ABI 7900HT Fast Real-Time PCR system (Applied Biosystems) based on the manufacturer’s guidelines. SpectroAquire and MassARRAY Typer Software (Sequenom) were used for interpretation, and the data were reviewed and analyzed using Typer analyzer (v3.4.0.18).

### Statistical analyses

All plasma markers were log_10_ transformed to achieve closer normality of the distributions. First, we examined demographic associations with blood pressure (BP) metrics and plasma biomarkers (GFAP, NfL, pTau181, Aβ42/40) via Spearman’s rank correlations or independent samples t-tests, as appropriate.

Multivariable linear regression models evaluated associations among blood pressure indicators (pulse pressure, systolic BP, diastolic BP) and plasma markers (GFAP, NfL, pTau181, Aβ42/40) each separately. All models covaried for age and sex. Given body size may influence blood volume levels and impact plasma biomarker measurement [37], we elected to partial out the effect of body mass index (BMI) on each plasma biomarker before entering the resulting residuals into regression models. Models evaluating plasma biomarkers without adjustment for BMI are available in Supplemental Table 1. To evaluate the influence of person-specific dementia risk factors, we added interaction terms to primary models testing the moderating role of *APOE-ε4* carrier status (ε4 carriers versus non-carriers), biological sex (female/male), or age on the relationship between blood pressure metrics and plasma biomarkers. Sensitivity models also evaluated the moderating effect of anti-hypertensive medication use (yes/no) on the relationship between BP metrics and plasma biomarker levels.

Across all models, effect sizes are reported as standardized betas and 95% CI or standard error.

## Results

Plasma concentrations of GFAP (*p* < 0.0001), pTau181 (*p* = 0.0008), and NfL (*p* < 0.0001) positively associated with age (Table 2). Lower levels of plasma Aβ42/40 weakly related to older age, though this association did not reach statistical significance (*p* = 0.30). Age was also associated with elevations in pulse pressure (*p* = 0.009), and approached but did not reach statistical significance for a positive association with systolic BP (*p* = 0.09). Diastolic BP demonstrated a negative association with age that approached significance (*p* = 0.09), which has been previously reported in older adults [38].

**Table 2:** Spearman’s rank correlations between age with plasma markers and blood pressure metrics

	Age	Plasma GFAP	Plasma NfL	Plasma pTau181	Plasma Aβ42/40	Systolic BP	Diastolic BP
Age							
Plasma GFAP	0.57*						
Plasma NfL	0.52*	0.60*					
Plasma pTau181	0.33*	0.33*	0.54*				
Plasma Aβ42/40	-0.10	0.08	-0.01	-0.01			
Systolic BP	0.17	0.05	0.16	0.29*	-0.0001		
Diastolic BP	-0.17	-0.28*	-0.18	-0.11	0.04	0.43*	
Pulse Pressure	0.255*	0.24*	0.27*	0.34*	-0.04	0.77*	-0.16

Note. *Statistically significant at *p* < 0.05

Diastolic BP differed by sex, such that males had higher diastolic blood pressure compared to females (Table 3). Systolic BP, pulse pressure, and plasma concentrations of Aβ42/40 and NfL did not statistically significantly differ by sex. However, females had statistically significantly higher concentrations of plasma GFAP compared to males, while males had a statistically significantly higher concentration of pTau181 than females (Table 3).

**Table 3:** Independent samples t-tests examining differences in demographic factors, blood pressure indicators and plasma markers by biological sex

	Males M(SD)	Females M(SD)	*p*-value
Age (years)	73.7(8.1)	72.8(8.2)	0.57
Education (years)	17.5(2.1)	17.5(1.9)	0.97
Systolic blood pressure (BP, mmHg)	136.0(14.3)	129.9(17.5)	0.053
Diastolic BP (mmHg)	76.4(9.7)	72.0(9.0)	0.02
Pulse pressure (systolic BP-diastolic BP, mmHg)	59.7(12.9)	57.9(15.1)	0.52
Amyloid beta 42/40 ratio (Aβ42/40)	0.06(0.001)	0.06(0.001)	0.46
Glial fibrillary acidic protein (GFAP)	149.3(66.0)	183.1(99.7)	0.04
Neurofilament light chain (NfL)	29.2(14.2)	29.9(15.1)	0.81
Tau phosphorylated at threonine-181 (pTau181)	4.7(2.5)	3.2(1.5)	0.0005

Note. *M* = mean, *SD* = standard deviation

Systolic and diastolic BP differed by anti-hypertensive medication use, such that the individuals prescribed anti-hypertensive medications had higher blood pressure readings (Systolic BP: mean = 139.1.±18.3; Diastolic BP: mean = 76.9.±10.2) compared to those who were not (Systolic BP: mean = 128.8.±14.0; Diastolic BP: mean = 72.4.±8.9; *p-value*s < 0.05). Pulse pressure did not differ based on anti-hypertensive medication use (Yes [medication use]: mean = 56.4.±12.9; No: mean = 62.2.±15.2; *p* > 0.05).

Plasma concentrations of GFAP, NfL, pTau181 and Aβ42/40 did not differ by *APOE*-ε4 status (*p*s > 0.05). Similarly, systolic BP, diastolic BP, and pulse pressure did not significantly differ based on *APOE*-ε4 carrier status (*p-values* > 0.05).

**Fig. 1:**
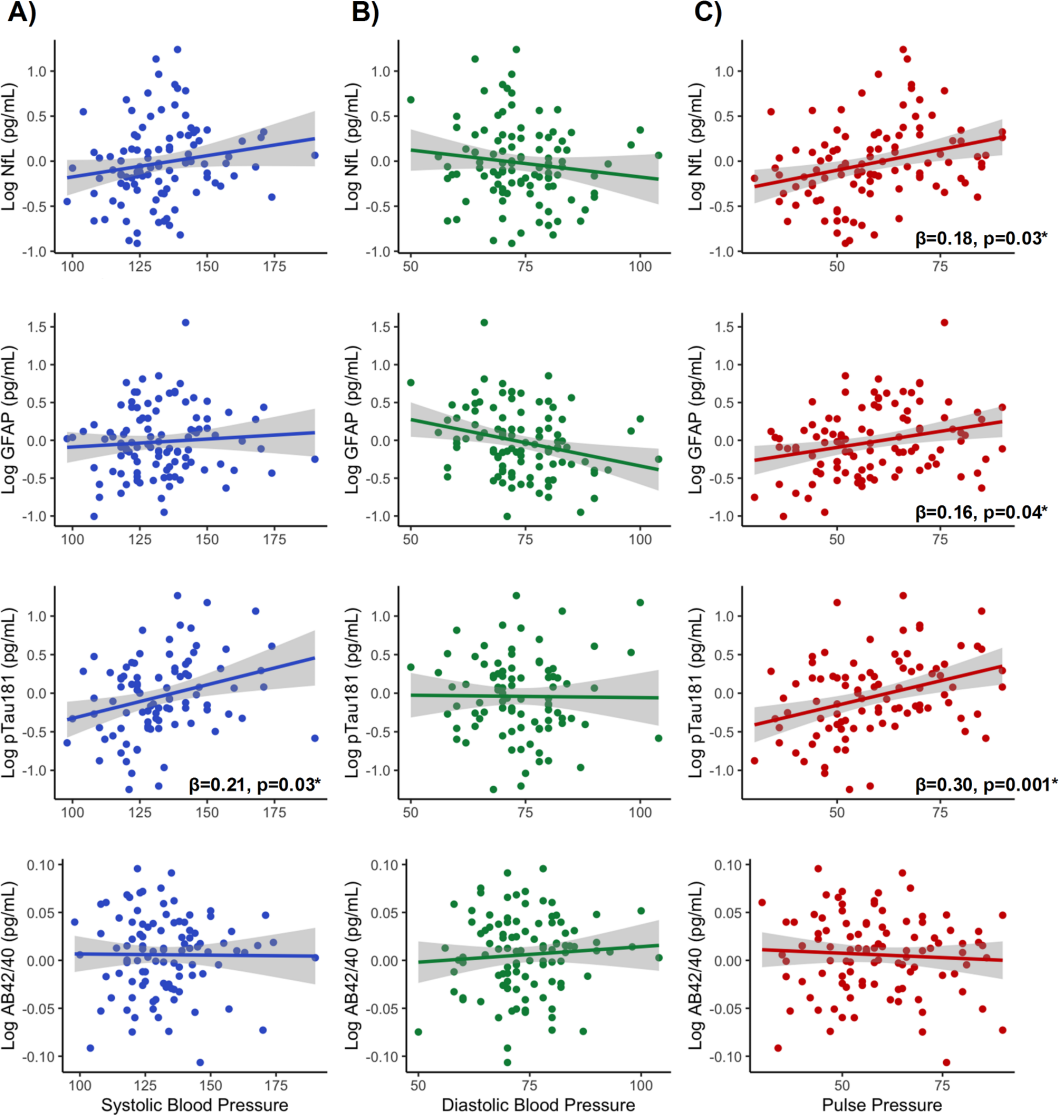
**a-c.** Multivariable linear regression models examining associations among blood pressure indicators and plasma markers, covarying for age and sex

Covarying for age and sex, elevated pulse pressure associated with higher concentrations of plasma pTau181, NfL, and GFAP, but not plasma Aβ42/40 (Fig. 1c; Table 4). Elevations in systolic BP were associated with higher plasma concentrations of pTau181, but did not statistically significantly relate to levels of Aβ42/40, GFAP, or NfL (Fig. 1a; Table 4). Diastolic BP did not meaningfully associate with any plasma biomarker concentrations, including Aβ42/40, GFAP, NfL, or pTau181 (Fig. 1b; Table 4).

**Table 4:** Multivariable linear regression models examining associations among blood pressure indicators and plasma markers

	Log GFAP		Log NfL		Log pTau181		Log Aβ42/40	
	β (95% CI)	*p*-value	β (95% CI)	*p*-value	β (95% CI)	*p*-value	β (95% CI)	*p*-value
**Age** **Sex** **Systolic BP**	0.56 (0.40, 0.72) 0.47 (0.14, 0.79) 0.06 (-0.10, 0.23)	< 0.001* 0.005* 0.45	0.51 (0.34, 0.68) 0.02 (-0.33, 0.36) 0.12 (-0.05, 0.30)	< 0.001* 0.93 0.155	0.22 (0.04, 0.41) -0.64 (-1.02, -0.27) 0.21 (0.02, 0.40)	0.02* 0.001* 0.03*	-0.13 (-0.33, 0.07) 0.09 (-0.31, 0.50) 0.01 (-0.19, 0.22)	0.22 0.65 0.90
**Age** **Sex** **Diastolic BP**	0.54 (0.38, 0.70) 0.38 (0.06, 0.71) -0.13 (-0.30, 0.03)	< 0.001* 0.02* 0.11	0.51 (0.34, 0.68) -0.06 (-0.41, 0.29) -0.06 (-0.23, 0.12)	< 0.001* 0.75 0.53	0.24 (0.04, 0.43) -0.74 (-1.13, -0.35) -0.06 (-0.26, 0.13)	0.02* < 0.001* 0.53	-0.11 (-0.31, 0.09) 0.12 (-0.29, 0.53) 0.07 (-0.13, 0.28)	0.27 0.55 0.49
**Age** **Sex** **Pulse Pressure**	0.52 (0.36, 0.69) 0.46 (0.15, 0.78) 0.16 (0.00, 0.32)	< 0.001* 0.004* 0.04*	0.48 (0.31, 0.65) -0.01 (-0.34, 0.32) 0.18 (0.01, 0.35)	< 0.001* 0.95 0.03*	0.18 (-0.01, 0.36) -0.69 (-1.05, -0.33) 0.30 (0.11, 0.48)	0.06 < 0.001* 0.001*	-0.12 (-0.32, 0.09) 0.09 (-0.32, 0.48) -0.03 (-0.24, 0.17)	0.26 0.67 0.75

Note. β = standardized beta values

### Interaction models

**Fig. 2:**
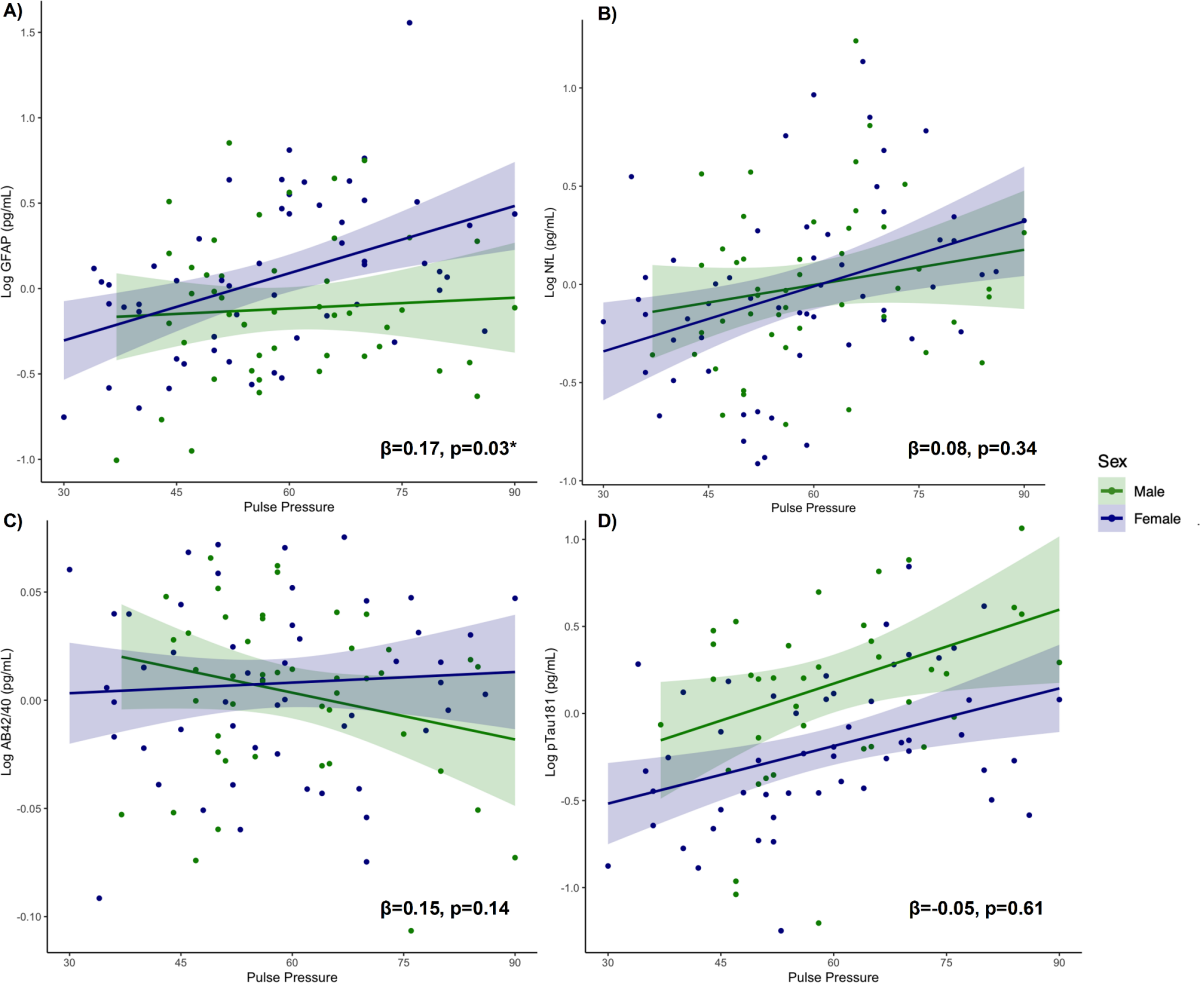
**a-d.** Interaction models examining whether associations between pulse pressure and plasma markers of interest differ by biological sex (male/female), covarying for age and sex

**Biological sex.** Interaction models revealed a significant interaction between biological sex and pulse pressure on GFAP concentrations (β = 0.17, *p* = 0.03), such that the association between elevated pulse pressure and higher plasma levels of GFAP was stronger in females (β = 0.30, *p* = 0.003) compared to males (β = 0.01, *p* = 0.81; Fig. 2a). Relationships between pulse pressure and plasma levels of NfL, pTau181, and Aβ42/40 did not differ based on biological sex (β_range_=-0.05-0.15, *p-values* > 0.14; Fig. 2b-d).

Similarly, the strength of associations between systolic BP and diastolic BP and each plasma biomarker did not significantly differ based on biological sex (β_range_=-0.12-0.11, *p-values* > 0.12).

**Fig. 3:**
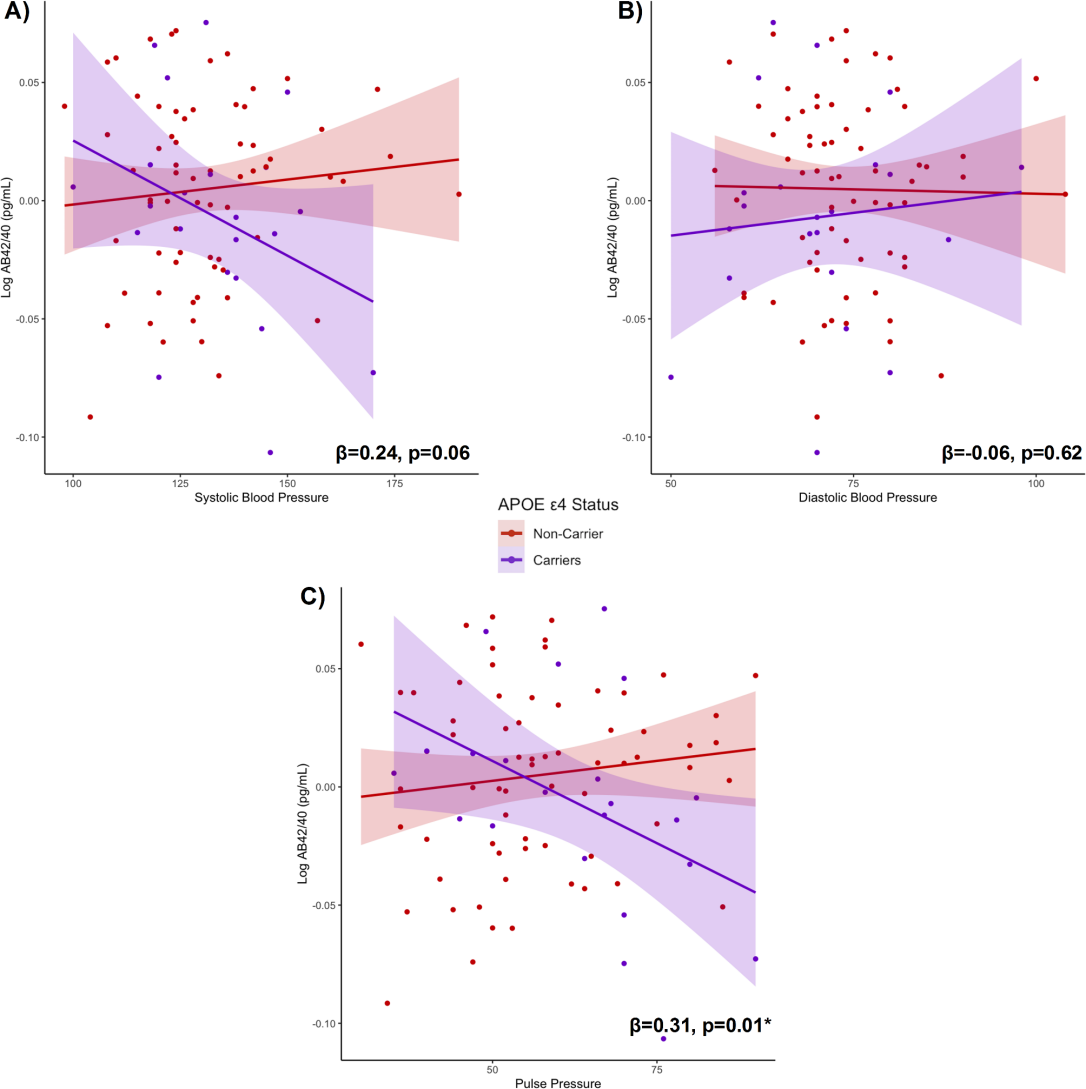
**a-c.** Interaction models examining whether associations among blood pressure indicators and plasma concentrations of Aβ42/40 differ based on *APOE*-ε4 status (ε4 carriers versus non-carriers), covarying for age and sex

***APOE*-ε4 status.** Next, we tested whether associations between blood pressure indicators and plasma markers differed based on *APOE*-ε4 status (ε4 carrier versus non-carrier). Indeed, there was a significant interaction between *APOE*-ε4 status and pulse pressure on Aβ42/40 concentrations (β = 0.31, *p* = 0.01), such that the association between elevated pulse pressure and lower plasma levels of Aβ42/40 was stronger among ε4 carriers (β=-0.43, *p* = 0.06) compared to non-carriers (β = 0.15, *p* = 0.24; Fig. 3c). Similarly, the interaction between *APOE*-ε4 status and systolic BP on Aβ42/40 approached statistical significance (β = 0.24, *p* = 0.06), while the relationship between diastolic BP and plasma levels of Aβ42/40 did not differ by *APOE*-ε4 status (β=-0.06, *p =* 0.62; Fig. 3a-b).

Interaction models revealed that the strength of associations between each BP indicator and plasma levels of GFAP, NfL, and pTau181 did not significantly differ based on *APOE*-ε4 status *(*β_range_=-0.01-0.12, *p-values* > 0.3).

**Age.** We also tested whether associations between blood pressure indicators and plasma markers differed based on age. Interaction models suggested that the strength of associations between blood pressure indicators and plasma biomarkers did not significantly differ based on age *(*β_range_=-0.11-0.14, *p-values* > 0.20).

**Anti-hypertensive medication use**. Sensitivity models evaluated whether the observed associations between blood pressure indicators and plasma markers differed based on whether participants were prescribed anti-hypertensive medications (yes/no). Interaction models suggested that the strength of associations between blood pressure indicators and plasma biomarkers did not significantly differ based on anti-hypertensive medication use *(*β_range_=-0.05-0.10, *p-values* > 0.20).

## Discussion

Our results demonstrate that systolic, diastolic, and pulse pressure metrics show differential relationships with molecular markers of neural aging among cognitively normal older adults. Specifically, elevated pulse pressure showed a consistently negative relationship with brain health evidenced across several key biomarkers (pTau181, NfL, GFAP), while elevated systolic blood pressure only associated with a marker of increased AD pathobiology (pTau181). Diastolic blood pressure did not significantly associate with any blood-based biomarker of brain aging. Post-hoc analyses revealed several person specific factors may influence the observed associations. Namely, elevated pulse pressure may be more important for astrocytic processes (GFAP) in females and for amyloid accumulation in *APOE*-ε4 carriers. It is important to note that while AD-related biomarker concentrations may not reflect fulminant AD pathology particularly at low levels, these markers may reflect early AD-related pathobiological processes. The clinical importance of these measures is further supported by their correlation with the clinical outcomes included in our study, suggesting that even at “subclinical” concentrations, these biomarkers may be indicative of heart to brain processes and have potential for early intervention [39–41]. Our study contributes to existing literature supporting the importance of cardiovascular health for healthy brain aging and further extends these findings by identifying which blood pressure metrics map onto established molecular markers of adverse brain aging. Furthermore, these data highlight the importance of blood pressure management, particularly in females and *APOE*-ε4 carriers, as a potentially high impact, scalable strategy for dementia prevention.

In particular, pulse pressure and systolic blood pressure demonstrated consistent, beneficial relationships with biomarkers of age-related neuronal health. Previous studies have demonstrated the importance of midlife blood pressure control, showing that plasma Aβ levels begin to decline at least 15 years before an AD diagnosis, with this decline linked to midlife systolic blood pressure [42]. Our study builds on these findings, highlighting a potentially ongoing important role of elevated systolic blood pressure and pulse pressure for neuronal markers of brain health in cognitively normal older adults. Elevated systolic blood pressure may directly impair glymphatic clearance of Aβ and pTau from the brain, contributing to neurodegenerative risk [43–45]. Moreover, previous research has proposed that elevated pulse pressure disrupts blood-brain barrier communication, which in turn triggers neuroinflammation and amyloid deposition, suggesting a possible mechanism through which pulse pressure may contribute to greater neuroinflammation, AD pathogenesis, and neurodegeneration as observed in our results [46]. On the other hand, while diastolic blood pressure is a marker of cardiovascular health, its direct relationship with neurodegeneration remains understudied. Existing research has primarily associated elevated diastolic blood pressure with impaired cognitive functioning but has not closely evaluated links with biomarkers of brain aging [47, 48]. Our study directly addressed this gap, with results suggesting that diastolic blood pressure may be a less important indicator of the brain changes evaluated compared to systolic or pulse pressure. This distinction between blood pressure metrics may be pathophysiologically significant. As previously discussed, diastolic blood pressure, reflecting vascular resistance and arterial elasticity, measures pressure during the heart’s resting phase [49, 50]. In contrast, systolic blood pressure reflects the force exerted during heart contraction and is more directly linked to arterial stiffness, cerebrovascular damage, and brain aging [51, 52]. Diastolic blood pressure may therefore have a weaker relationship with these processes, as it does not capture the pulsatile forces or strain impacting cerebral vasculature, which may explain its reduced significance in predicting brain aging in our study [53]. However, future work is needed to determine the robustness of this null finding.

Given AD pathobiology disproportionately affects females compared to males, sex-specific differences might be expected in our analyses [54]. However, the relationships between blood pressure metrics and primary AD pathobiology markers did not differ based on biological sex. Additionally, the male predisposition towards higher rates of cardiovascular disease might suggest the presence of male-specific differences in the relationships between blood pressure and plasma biomarkers of neuronal aging [26]. Instead, we found that elevated pulse pressure was associated with higher levels of astrocytic activation (GFAP) in females only. No sex-based differences were noted in the associations of systolic and diastolic blood pressure or with any other biomarkers beyond GFAP. Thus, our study suggests that sex differences in systemic cardiovascular health may primarily relate to neuroinflammatory outcomes in older females [55]. This relationship may be explained through shifts in cardiovascular and immune risk following menopause, which may reduce the known disparity in cardiovascular health between males and females. Indeed, postmenopausal females show elevated cardiovascular risk following significant declines in estrogen over the menopausal transition, including increased arterial stiffening [56], and estrogen is a regulator of immune homeostasis. Astrocytes play a central role in the brain’s immune response; our data may therefore be consistent with heightened astrocytic activation in postmenopausal females that is at least in part linked to increased cardiovascular risk following decreases in estrogen. This hormonal shift may enhance astrocyte responsiveness to cardiovascular stress, leading to greater involvement of neuroinflammatory pathways, thereby supporting the pronounced association with GFAP in females. More work directly linking sex specific biology, such as the menopause transition, sex hormones, and X chromosome expression, with cerebrovascular and glial function is needed to more fundamentally understand these relationships.

We further emphasize the importance of understanding genetic predispositions in relation to cardiovascular health and its influence on neuronal aging biomarkers. We found a unique association between elevated pulse pressure and lower Aβ42/40 concentrations in *APOE*-ε4 carriers. A similar, though marginally significant, trend was observed for systolic blood pressure. In contrast, diastolic blood pressure did not show a differential effect based on *APOE*-ε4 status. These results suggest that poorly managed pulse pressure or blood pressure may *disproportionately* increase risk for Aβ in *APOE*-ε4 carriers. Our results align with previous literature that suggests *APOE*-ε4 carriers have increased risk of Aβ accumulation [57]. However, the underlying mechanism connecting *APOE*-ε4 to Aβ is unclear. It is possible the *APOE*-ε4 polymorphism leads to impaired clearance of Aβ, increased production of Aβ, or a combination of both. However, there may be other indirect biological mechanisms, such as disrupted blood-brain barrier integrity, which has been previously observed in *APOE*-ε4 carriers, and may in turn exacerbate Aβ dysregulation [58]. Although the exact mechanisms are not fully understood, our findings suggest that poor management of pulse pressure in individuals with this genetic risk factor may contribute to Aβ dysregulation. Of note, we did not find an effect of age on relationships between blood pressure and neural biomarkers, which may suggest that blood pressure influences neural biomarkers uniformly across the older adult lifespan quantified in this study (range: 50 to 90 years old). Alternatively, this result could be due to limited statistical power or the possibility that age-related variations in these relationships are more nuanced than initially hypothesized. Our work reinforces the importance of considering person-specific factors and clinical context highlighting potential benefits of interventions aimed at managing blood pressure to mitigate the risk or progression of neurodegenerative diseases, especially in those with genetic vulnerabilities.

### Limitations

Our study has many strengths including the use of high-sensitivity assays, ensuring precise measurement of our blood biomarkers, and bolstering the reliability of the findings and advancing our understanding of cardiovascular-brain health dynamics. While our work is novel in approach and reveals important insights into the role of cardiovascular health in dementia risk pathways, our study is not without limitations. While we used some of the most widely available biomarkers on one of the most common platforms (i.e., Quanterix), we recognize the field is rapidly evolving and other methods of quantifying these biomarkers are available, thus it will be important to continue to test these across platforms and assays (e.g., C2N, pTau-217). A limitation of our study is the potential lack of precise Aβ42/40 quantification, as more advanced assays, such as mass spectrometry, offer greater accuracy [59]. However, certain immunoassays, including those utilizing the Simoa platform, strongly correlate with mass spectrometry-based methods and remain widely used due to their practicality [60]. Moreover, we acknowledge that there are other factors that can impact biomarker concentrations (e.g., chronic kidney disease, liver disease) and we did not have the data to account for the possible impact of these other organ systems on biomarker outcomes [61, 62]. Additionally, our sample was 79% White with high average educational attainment which may limit generalizability of study findings. Lastly, the study’s observational design restricts the ability to draw causal conclusions about the examined relationships. While the study highlights associations, it does not fully elucidate underlying biological mechanisms, and the relatively small sample size of healthy older adults limits statistical power, which may impact the detection of interactions, suggesting further research is needed to inform precise interventions.

## Conclusions

We found that elevated pulse pressure was the most sensitive metric, negatively associated with axonal degeneration, astrocytic activation, and AD pathobiology (NfL, GFAP, pTau181), while elevated systolic blood pressure associated with increasing pTau burden (pTau181). These findings highlight possible pathways that may be affected by poorly managed blood pressure for brain health. Unlike previous studies using composite cardiovascular risk scores, we examined individual cardiovascular indicators specific to blood pressure, offering insights into preventive strategies and more specific interventions to safeguard cognitive function and reduce neurodegenerative disease risk. We further contribute to the body of evidence showing physiological associations between blood pressure and brain health, highlighting blood pressure as a pivotal intervention target. Moreover, tailoring interventions based on individual risk profiles may support more potent dementia prevention and management approaches, ultimately improving patient outcomes. Future research should continue to leverage novel fluid biomarker tools, such as unbiased proteomics, to more deeply understand the complexities of the cardiovascular to brain connection and further, to identify specific targets and pathways that mediate the effects of cardiovascular health, such as blood pressure on brain function.

## Electronic supplementary material

Below is the link to the electronic supplementary material.

Supplementary Material 1

## Data Availability

The dataset used and analyzed for the current study are available from the corresponding author on reasonable request.

## Abbreviations

AD: Alzheimer’s disease
BP: Blood pressure
pTau: Phosphorylated tau
Aβ: Beta amyloid
NfL: Neurofilament light chain
GFAP: Glial fibrillary acidic protein
Aβ42/40: Amyloid beta 42/40 ratios
*APOE*-ε4: Apolipoprotein ε4
BMI: Body mass index
CDR scale: Clinical dementia rating scale
pTau181: Tau phosphorylated at threonine-181
