# Supplementary material for: Relationships between blood pressure indicators and fluid biomarkers of brain aging in functionally intact older adults
Source: Alzheimers Res Ther. 2025 Apr 21;17:85. doi: 10.1186/s13195-025-01731-9 (PMC12010523; doi:10.1186/s13195-025-01731-9)
Supplement: Supplementary file 1 — Supplementary Material 1 [file 13195_2025_1731_MOESM1_ESM.docx]

**Supplemental Table 1.** Multivariable linear regression models examining associations among blood pressure indicators and plasma markers without the effects of BMI regressed out.

|  | **Log GFAP** | | **Log NfL** | | **Log pTau181** | | **Log Aβ42/40** | |
| --- | --- | --- | --- | --- | --- | --- | --- | --- |
|  | **β (95% CI)** | **p-value** | **β (95% CI)** | **p-value** | **β (95% CI)** | **p-value** | **β (95% CI)** | **p-value** |
| Age  Sex  Systolic BP | 0.57 (0.41, 0.72)  0.48 (0.16, 0.80)  0.02 (-0.14, 0.19) | <0.001*  0.004*  0.78 | 0.52 (0.35, 0.69)  0.04 (-0.30, 0.39)  0.07 (-0.11, 0.24) | <0.001*  0.80  0.45 | 0.24 (0.05, 0.42)  -0.64 (-1.02, -0.26)  0.18 (-0.01, 0.37) | 0.01*  0.001*  0.057 | -0.13 (-0.33, 0.07)  0.09 (-0.31, 0.50)  0.01 (-0.19, 0.22) | 0.21  0.65  0.89 |
| Age  Sex  Diastolic BP | 0.54 (0.38, 0.70)  0.40 (0.08, 0.72)  -0.16 (-0.32, 0.00) | <0.001*  0.02*  0.06 | 0.51 (0.34, 0.69)  -0.02 (-0.37, 0.32)  -0.09 (-0.27 0.08) | <0.001*  0.90  0.30 | 0.24 (0.05, 0.44)  -0.73 (-1.13, -0.34)  -0.08 (-0.28, 0.12) | 0.01*  <0.001*  0.41 | -0.11 (-0.31, 0.09)  0.12 (-0.29, 0.53)  0.07 (-0.13, 0.28) | 0.27  0.55  0.48 |
| Age  Sex  Pulse Pressure | 0.54 (0.38, 0.70)  0.48 (0.17, 0.80)  0.13 (-0.03, 0.29) | <0.001*  0.002*  0.10 | 0.50 (0.33, 0.67)  0.04 (-0.30, 0.37)  0.14 (-0.03, 0.31) | <0.001*  0.83  0.11 | 0.19 (0.01, 0.38)  -0.68 (-1.04, -0.31)  0.28 (0.09, 0.46) | 0.04*  <0.001*  0.004* | -0.12 (-0.32, 0.09)  0.08 (-0.32, 0.48)  -0.03 (-0.24, 0.17) | 0.26  0.68  0.75 |

Note. β = standardized beta values.
